# Phonon and magnetic structure in δ-plutonium from density-functional theory

**DOI:** 10.1038/srep15958

**Published:** 2015-10-30

**Authors:** Per Söderlind, F. Zhou, A. Landa, J. E. Klepeis

**Affiliations:** 1 Lawrence Livermore National Laboratory, Livermore, CA 94550, USA

## Abstract

We present phonon properties of plutonium metal obtained from a combination of density-functional-theory (DFT) electronic structure and the recently developed compressive sensing lattice dynamics (CSLD). The CSLD model is here trained on DFT total energies of several hundreds of quasi-random atomic configurations for best possible accuracy of the phonon properties. The calculated phonon dispersions compare better with experiment than earlier results obtained from dynamical mean-field theory. The density-functional model of the electronic structure consists of disordered magnetic moments with all relativistic effects and explicit orbital-orbital correlations. The magnetic disorder is approximated in two ways: (i) a special quasi-random structure and (ii) the disordered-local-moment method within the coherent potential approximation. Magnetism in plutonium has been debated intensely, but the present magnetic approach for plutonium is validated by the close agreement between the predicted magnetic form factor and that of recent neutron-scattering experiments.

The properties of plutonium metal are rather extraordinary with perhaps the most striking being its ambient pressure phase diagram^1^. The fact that on one hand the material exists in a very low symmetry and high density monoclinic α phase and on the other in a high symmetry and low density cubic δ phase is remarkable and unlike any other condensed matter system. To make plutonium even more intriguing, there are four more phases (β, γ, δ’, and ε) before melt as shown in Fig. 1.

The plutonium phase diagram is of course a great challenge for theory; consider its lighter cousin cerium and its *one* isostructural phase transition (α to γ) that is still a focus of debate, experimentation, and modeling^2^. Nonetheless, the Letter by Söderlind and Sadigh^3^ clearly showed that the main features of the plutonium phase diagram could be understood in terms of itinerant (delocalized) 5*f* electrons that support formation of magnetic moments. In a series of papers^3^,^4^,^5^,^6^ the authors explain that the magnetic moments must be disordered in the δ phase because any magnetic order is mechanically destabilizing. This conclusion naturally answers why δ-plutonium does not exist at lower temperatures below the magnetic ordering temperature. Another piece of the puzzle was resolved when it was realized^7^,^8^ that the spin and orbital moments cancel each other, rendering δ-Pu effectively nonmagnetic. Furthermore, the spin-polarized electronic structure shows good agreement with photo emission spectra^9^.

There has been some criticism^10^ of the theory for plutonium because of its prediction of magnetic moments. It was pointed out that neutron-scattering data showed no evidence of ordered or disordered moments and it was argued that magnetic moments are completely absent in plutonium^10^. Because of a new development in experimental measurements on plutonium we will return to this issue below.

In spite of the critique regarding plutonium magnetism, it is clear from the phase-diagram^3^ and elastic-constant calculations^11^ that the DFT total energies are accurate and reliable for plutonium metal. Hence, we are here taking advantage of this fact in applying an advanced scheme to compute plutonium lattice dynamics from first-principles theory. The compressive sensing lattice dynamics (CSLD) method determines force constants and lattice dynamics and requires only total-energy calculations as input^12^. We utilize the robust and mathematically rigorous framework of compressive sensing (CS), a new technique in the field of information science for recovering sparse solutions from incomplete data^13^, to resolve which harmonic or anharmonic terms are important and simultaneously find their values. From CSLD we calculate phonon dispersions for δ-plutonium utilizing two separate implementations of DFT that we describe in the modeling section below. The most important difference between them is their treatment of magnetic disorder, which is modeled by either a special quasi-random structure (SQS) or the disordered-local-moment (DLM) method within the coherent-potential approximation (CPA). The latter approach can easily be extended to also model solid solutions of δ-Pu-Ga or other alloys.

The Results section presents the calculated phonon dispersions and makes contact with existing experimental data and other theoretical modeling. In the Discussion section we return to the issue of magnetism in plutonium and compare the previously calculated magnetic form factor with that measured very recently by neutron scattering. Lastly, in the Methods section we detail the electronic-structure calculations as well as aspects of our lattice-dynamics approach for the δ-plutonium phonons.

## Results

Assuming that the Taylor expansion^12^ that is fitted to the total energies in the CSLD scheme is converged, we calculate the lattice dynamics corresponding to our two DFT approaches, FPLMTO and EMTO (see definitions in the Methods section). Because the former method provides the more accurate electronic structure, with the caveat that magnetic disorder may be better modeled within EMTO, we focus first on the results from FPLMTO.

**Figure 2:**
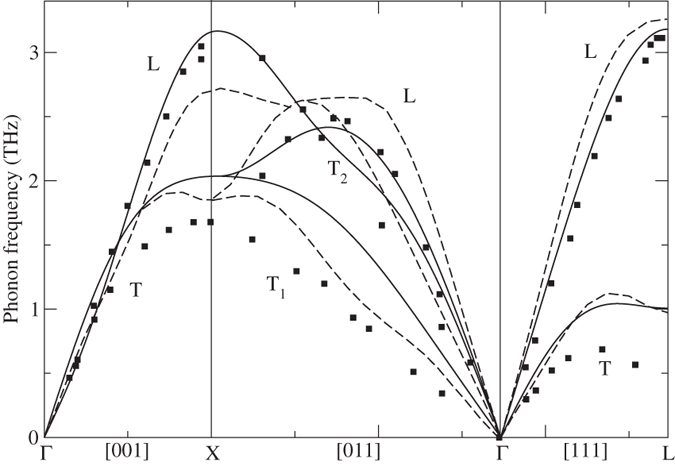
FPLMTO-CSLD (solid line), DMFT (dashed line)^15^, and experimental^14^ phonons for δ-plutonium.

In Fig. 2 we show the FPLMTO-CSLD phonon dispersions for δ-Pu together with experimental data from Wong *et al.*^14^ and results obtained using the dynamical mean-field theory (DMFT)^15^. Our results (solid line) generally agree quite well with inelastic x-ray scattering^14^, particularly all longitudinal branches. The slopes of the Γ-X [001] longitudinal (L) and transverse (T) branches at the Γ point correspond to the c_11_ and c_44_ elastic moduli, while the slope at the Γ point of the T_1_ branch along Γ-X [011] relates to c’ = ½(c_11_ − c_12_). A close inspection of Fig. 2 reveals that our calculations slightly overestimate c_11_ and c’, while c_44_ and c_12_ are in close agreement with experiment. This was also the conclusion from our previous investigation of the elastic moduli for δ-plutonium^11^. Furthermore, in Fig. 2 we are able to compare our DFT results (solid line) with those of DMFT (dashed line)^15^. The authors of^15^ state that DFT “has limited applicability” but in reality the DFT results agree better with the experimental data^14^ than those obtained from DMFT.

**Figure 3:**
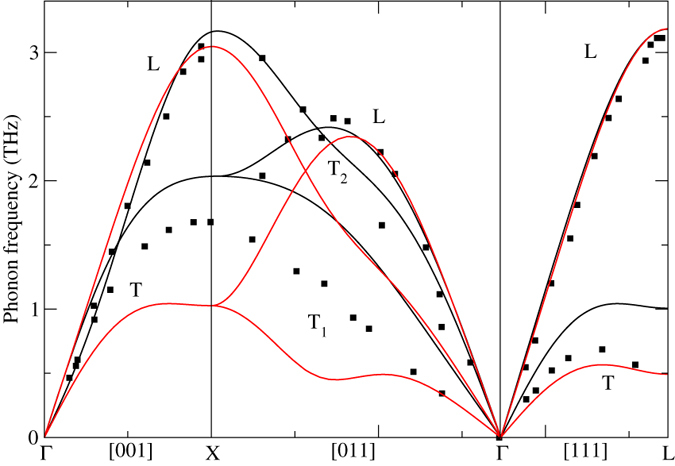
FPLMTO-CSLD (solid line), EMTO-CSLD (red line), and experimental^14^ phonons for δ-plutonium.

In Fig. 3 we show the lattice dynamics obtained from EMTO total energies combined with CSLD. The EMTO method predicts somewhat larger zone-boundary phonons than FPLMTO^16^ and for an easier comparison we scale the EMTO phonon frequencies so that the L-point L phonon coincides. After this scaling all high-energy phonons agree very well between the two methods but for some of the softer transverse phonons there are differences. It is also for these softer phonons that both the FPLMTO and DMFT results diverge from the experimental measurements by Wong *et al.*^14^. One reason for this discrepancy may be that the sample used for the inelastic x-ray scattering was a δ-Pu-Ga alloy, while all theoretical work thus far has modeled unalloyed δ-Pu. A sophisticated investigation of the δ-Pu-Ga alloy system, applying the EMTO-CPA and CSLD methods, is forthcoming and may resolve this speculation.

## Discussion

We have shown that combining a new, efficient scheme for lattice dynamics with density-functional theory gives quite good phonon properties for δ-plutonium that agree better with inelastic x-ray scattering than those predicted by dynamical mean-field theory. The DFT-CSLD methodology has distinct advantages over DMFT in that it is entirely parameter free and it easily couples to advanced modeling of alloys via the CPA. The presented results suggest that realistic predictions of lattice dynamics are possible for plutonium alloy systems such as the Pu-Ga-types that are primarily used in the measurements.

The critique against DFT for plutonium has generally been that no magnetic moments are known to exist thus contradicting the theory. The assumption that magnetic moments are absent in plutonium is reasonable based on the body of experimental evidence against their presence^10^. However, it is equally reasonable that the complex magnetism predicted by DFT has been too elusive or complex to recognize experimentally. Certainly the magnetic disorder and cancellation of spin and orbital moments make the magnetism obscure for most experimental probes. Lander^17^ agrees that anti-parallel spin and orbital moments represent a more complex situation but argues in ref. 10 that even in the complete cancellation scenario, where the total magnetic moment is equal to zero, “the difference in their [spin and orbital] spatial extent would still allow a measureable signal to be seen in neutron scattering”. Apparently no such signal was detected in neutron-scattering work up to that point^10^.

It is actually rather straightforward to calculate the neutron-scattering signal in terms of the magnetic form factor, at least within the dipole approximation. From the band-structure computation one obtains spin- and orbital-moment densities and then the magnetic form factor (magnetic scattering amplitude) is calculated as a Fourier transform of the magnetization density


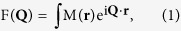


where **Q** is the scattering vector. In the dipole approximation^18^ it can be expressed as


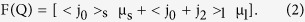


Here Q = |**Q**| and <j_n_> are averages of Bessel functions over the plutonium-atom spin density and μ_s_ and μ_l_ are magnetic spin and orbital moments, respectively. In the cancellation model^8^ these moments are anti-parallel with the same magnitude so that μ_s_ + μ_l_ = 0. In this special case, F reduces to a scaled j_2_ function and one therefore expects the magnetic form factor to behave like j_2_ with a shoulder at finite Q vector and to vanish at Q = 0. This behavior has been observed for α-Sm where the magnetic cancellation is nearly complete^19^.

**Figure 4:**
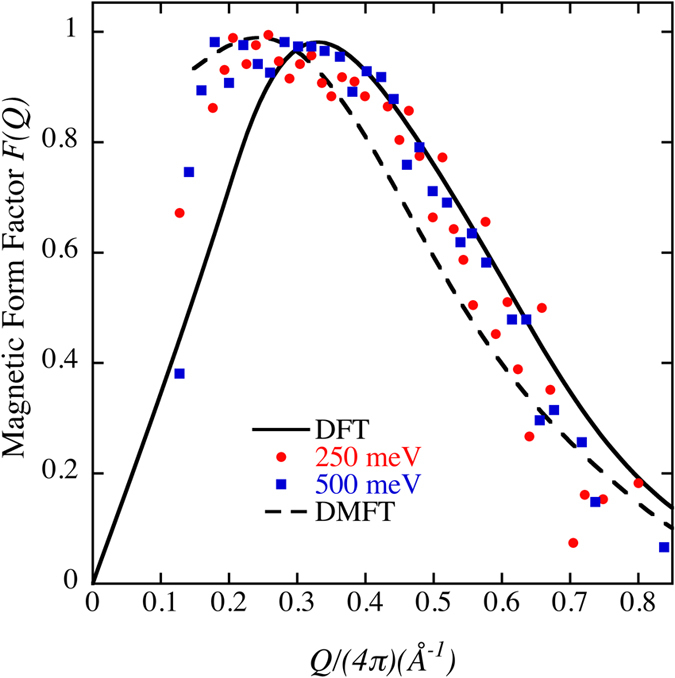
DFT (solid line)^8^, neutron-spectroscopy (solid symbols)^20^, and DMFT (dashed line)^20^ magnetic form factor for δ-plutonium.

In Fig. 4 we show the DFT prediction^8^ of the magnetic form factor (solid line) that shows the behavior of the j_2_ Bessel function. In this figure we also plot very recent magnetic form factor data for δ-Pu obtained from neutron-spectroscopy experiments with two incident neutron energies (250 and 500 meV)^20^. Most of the error bars on the experimental data in the original plot (Fig. 2 in^20^) are small and are removed here for clarity. There is obviously very good agreement between the measurement and the prediction from DFT made some years ago^8^. Two important features of the DFT and the experimental data are that both have a shoulder close to Q ~ 0.25–0.3 and they both approach zero for smaller Q values. This behavior indicates a very efficient destruction of the net total magnetic moment in δ-Pu because at Q = 0 the value corresponds to the magnetic moment integrated over the full crystal. The DMFT model^20^ agrees almost as well with the neutron-spectroscopy data but seems to lack the correct functional form for small Q.

Janoschek *et al.*^20^ make the point that spin moments do exist in δ-Pu, contrary to the conclusion by Lashey *et al.*^10^, and that they are fluctuating by means of valence fluctuations. This conclusion is consistent with our DFT results because magnetic disorder simply represents a frozen (static) state of the fluctuations. One important difference in the DMFT interpretation^20^ of the measurements and the present theory is the description of the 5*f* electrons. The DMFT^20^ models the 5*f*-electron states as superposition of localized 5*f* wave functions with screened magnetic moments while DFT describes the 5*f* electrons as itinerant with spin and orbital moments effectively cancelling each other. The behavior of the 5*f* electrons is essential for the character of the chemical bonding which is different for the two (DFT and DMFT) models. Eriksson *et al.*^21^ proposed an approach that captures features of both these models where a portion of the 5*f* manifold is localized. Importantly, the delocalization of 5*f* electrons provides appropriate attractive bonding that not only explains the atomic volume for the δ phase but the volumes for all the other phases as well^3^. Conversely, the DMFT interpretation cannot explain the lattice constant for δ-Pu (nor any other phases) because localized 5*f* electrons do not provide sufficient bonding. This conclusion has been supported by spin polarized and strongly correlated (GGA + Hubbard U) calculations that severely overestimate the atomic volume for δ-Pu^22^,^23^.

The lack of bonding for localized *f* states leads to larger volumes and phase diagrams that are nearly invariant with the number of *f* electrons and these facts have been discussed in detail for both rare-earth^24^ and actinide^25^,^26^,^27^,^28^ metals.

## Methods

Within the density-functional-theory approach one important and necessary assumption is the choice of the electron exchange and correlation functional. Because of the success of the generalized-gradient approximation (GGA) for actinide metals in general^29^ it is applied here for the δ-plutonium electronic structure. We are employing two separate implementations of DFT-GGA for the calculations of the total energies required for the CSLD. First, we utilize the full-potential linear muffin-tin orbital method (FPLMTO)^30^ for the best possible accuracy of energetics related to atomic displacements. Second, the exact muffin-tin orbital (EMTO) methodology^31^ is applied similarly, but with somewhat lesser accuracy with respect to atomic displacements. However, the EMTO method takes advantage of the CPA that allows for easy modeling of magnetic as well as atomic disorder.

The FPLMTO method has been tested thoroughly for plutonium metal^3^,^11^ and most of the details of the present calculations replicate that of Söderlind and Sadigh^3^. The magnetic disorder for δ-Pu in^3^ was accomplished by an eight atom special quasi-random structure, while here we are applying a 32-atom cell for this purpose, similar to what was used for paramagnetic face-centered-cubic iron in the report by Körmann *et al.*^32^. The spin-orbit interaction and orbital polarization are treated exactly as in^3^ except that the orbital polarization correction is here only done for the 5*f* states (not also the 6*d* states). The difference is less important because the orbital moments from the 6*d* states are small (~0.1 μ_B_). Because of the application of random displacements of the atoms, necessary for the CSLD, the crystal has no symmetry and a total of 32 k points are utilized for the electronic structure. All FPLMTO calculations are carried out at the theoretical equilibrium lattice constant (4.635 Å) that is very close to the established handbook data (4.637 Å)^33^.

The details of the EMTO computations are identical to those presented in an earlier report^16^. Here they are performed for a 64-atom super-cell at the EMTO equilibrium lattice constant (4.669 Å). Notably, spin-orbit coupling is not included while the spin disorder is that of a paramagnetic disordered-local-moment model^34^. This state uses a random mixture of two distinct magnetic states (spin up and down) on the same atomic species (Pu).

To train the CSLD model and compute harmonic force constants for δ-Pu, total-energy calculations are performed for super-cells with (1) random atomic displacements between 0.05–0.26 Å and (2) a few select frozen-phonon configurations. The total number of configurations is rather large, about 250 and 150 for FPLMTO and EMTO, respectively.

For the FPLMTO-SQS configurations, we apply relatively large displacements to avoid the mechanical instability that may occur for any particular SQS arrangement. The second (harmonic), third, and fourth order force constants of the lattice are taken into account in the CSLD fitting, resulting in 109 independent parameters after considering constraints on the forces due to crystal symmetry and translational invariance^12^. By considering the anharmonic (third and fourth) order force constants the fit is accurate even though the displacements are relatively large. We divide the training data into a fitting and a prediction (validation) subset, obtaining CSLD force constants with the fitting data and then computing the error on the prediction subset at a given μ parameter^12^,^35^. An average is then calculated by repeating the above procedure 10 times and the optimal force constants are obtained by minimizing the prediction error (~6%) over μ.

## Additional Information

**How to cite this article**: Söderlind, P. *et al.* Phonon and magnetic structure in δ-plutonium from density-functional theory. *Sci. Rep.* **5**, 15958; doi: 10.1038/srep15958 (2015).

## Figures and Tables

**Figure 1:**
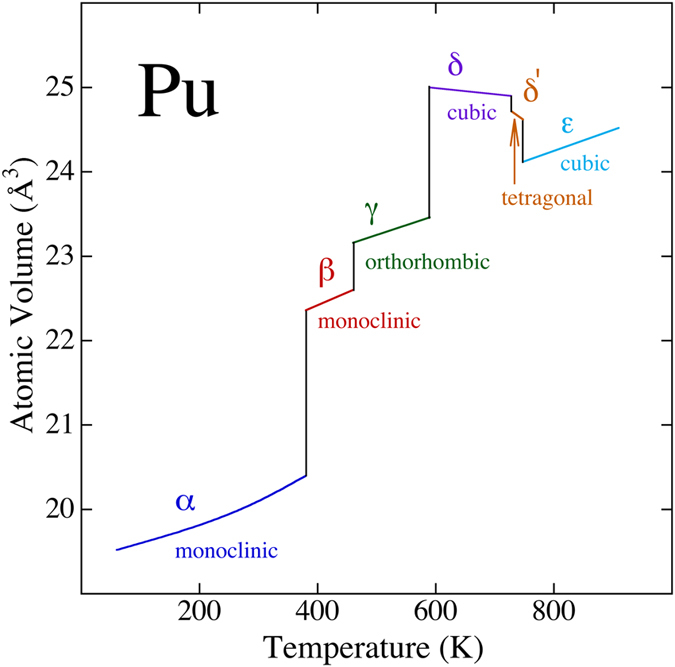
The experimental phase diagram for plutonium metal. Redrawn after^1^.
